# Flexible Fabrication and Hybridization of Bioactive Hydrogels with Robust Osteogenic Potency

**DOI:** 10.3390/pharmaceutics15102384

**Published:** 2023-09-26

**Authors:** Liang Zhu, Qian Hou, Meijun Yan, Wentao Gao, Guoke Tang, Zhiqing Liu

**Affiliations:** 1 Department of Orthopedics, Zhuzhou Hospital Affiliated to Xiangya School of Medicine, Central South University, Zhuzhou 412001, China; zhuliangspine@hotmail.com (L.Z.); nhdxgwt@126.com (W.G.); 2 Department of Orthopedics, Shanghai General Hospital, Shanghai Jiaotong University, Shanghai 200080, China; yanmeijun1105@163.com; 3 Department of Nutrition, Xiangya Hospital, Central South University, Changsha 410008, China; qhou2005@163.com

**Keywords:** bioactive hydrogels, adhesive, osteogenesis, tetra-PEG, bioactive glass

## Abstract

Osteogenic scaffolds reproducing the natural bone composition, structures, and properties have represented the possible frontier of artificially orthopedic implants with the great potential to revolutionize surgical strategies against the bone-related diseases. However, it is difficult to achieve an all-in-one formula with the simultaneous requirement of favorable biocompatibility, flexible adhesion, high mechanical strength, and osteogenic effects. Here in this work, an osteogenic hydrogel scaffold fabricated by inorganic-in-organic integration between amine-modified bioactive glass (ABG) nanoparticles and poly(ethylene glycol) succinimidyl glutarate-polyethyleneimine (TSG-PEI) network was introduced as an all-in-one tool to flexibly adhere onto the defective tissue and subsequently accelerate the bone formation. Since the N-hydroxysuccinimide (NHS)-ester of tetra-PEG-SG polymer could quickly react with the NH_2_-abundant polyethyleneimine (PEI) polymer and ABG moieties, the TSG-PEI@ABG hydrogel was rapidly formed with tailorable structures and properties. Relying on the dense integration between the TSG-PEI network and ABG moieties on a nano-scale level, this hydrogel expressed powerful adhesion to tissue as well as durable stability for the engineered scaffolds. Therefore, its self-endowed biocompatibility, high adhesive strength, compressive modulus, and osteogenic potency enabled the prominent capacities on modulation of bone marrow mesenchymal stem cell (BMSCs) proliferation and differentiation, which may propose a potential strategy on the simultaneous scaffold fixation and bone regeneration promotion for the tissue engineering fields.

## 1. Introduction

Bone tissue has an inborn and unique ability of self-regeneration for many variously minor injuries, but some serious diseases like bone fracture defects, trauma, and even osteosarcoma are still dangerous in orthopedics [1,2,3]. Although there has been significant progress in the development of surgical techniques, the operation difficulty and the incidence of delayed healing or nonunion will eventually result in the disability and significant socio-economic burden in clinical practice. Scientists and doctors are trying to improve the surgical treatment of autologous and allograft bone grafting above these severe bone defects, but it may inescapably lead to clinical complications of donor shortage, delayed operation, limited donor volume, donor site immune rejection, and probably bacterial infection [4,5,6,7,8]. In view of these shortcomings, the emergence of tissue engineering technology, taking advantage of exogenous progenitor cells and the controlled release of biotic components, has provided innovative ideas and promising methods for promoting bone regeneration [9,10,11,12,13,14].

The rapid development of hydrogel scaffolds with preferred biocompatibility, sufficient mechanical strength, matching degradation rate, and promotional osteogenic activity has become a hot spot in bone tissue engineering research [15,16,17,18,19,20]. Hydrogels, mainly including synthetic hydrogels and natural hydrogels, are generally composed of three-dimensional networks with multifarious pores and polymeric structures, which can be widely used for drug release systems, tissue engineering fields, and flexibly intelligent materials [21,22,23,24]. Despite the great progress that has been achieved in the development of hydrogels over the past few decades, the respective insufficiency of weak strength of naturally-derived hydrogels and poor biocompatibility of synthetic hydrogels remains the main challenge and constraint on acquiring the hydrogel scaffolds in clinic [25,26,27,28]. Therefore, increasing the mechanical strength of natural polysaccharides, improving the cell compatibility of synthetic polymers, or integrating their advantages on preparing the multifunctional composite hydrogels are mainly methods to explore the tissue-engineered hydrogels in biomedical applications.

Branched polymers with dendritic structures and abundant terminal groups have been an ideal crosslink to design the complex networks and advanced functions for the topological polymers and hydrogels [29,30,31,32]. Much of the research to date involves the simple crosslinking between the branched polyethyleneimine (PEI) and another polymers such as polyacrylic acid (PAA), poly(vinyl alcohol), and poly(ethylene glycol) via the multiply electrostatic interactions within a few seconds [33,34,35,36,37]. For example, the resulting PEI/PAA hydrogel scaffold exhibits preferred cell biocompatibility and powerful self-healing ability, but the bone conduction and other biological properties are required for the further modifications [37]. Therefore, incorporation of the bioactive components or biological factors is an effective approach to improving the bio-function of tissue-engineered constructs.

Bioactive glass (BG) comprised of silicon, calcium, and phosphorus elements has advanced the ability to induce bone mineralization to repair bone defects. Especially under physiological conditions, BG can produce a hydroxyapatite layer through chemical combination with bone tissue, similar to the stage of forming bone mineral, and provides a favorable environment for the activity, growth, proliferation, and differentiation of osteoblast-related cells [38,39]. However, the complex microenvironment always leads to the microphase separation between inorganic phases and organic networks that can induce the structural heterogeneity, endanger the stability, and impair the osteogenic potency of the BG-based composites, ultimately leading to an imbalance between bone growth and degradation of the implanted material [40,41]. Considering the decisive role of ensuring the stably osteogenic activity, BG particles were necessarily required for the further physical–chemical modification. For example, Chen et al. [42] proposed a modified composite strategy on preparing amine-terminated bioactive glass (ABG) particles that were incorporated into the adhesive glue on the nanoscale level. Along with the gradual hydrogel degradation in vivo, ABG was expose-contacted and subsequently bound onto the bone tissues to facilitate angiogenesis, which exhibited the beneficial nanophase integration, bone mineralization, mechanical strength, adhesion force, and osteo-activation for in vivo bone regeneration.

**Figure 1:**
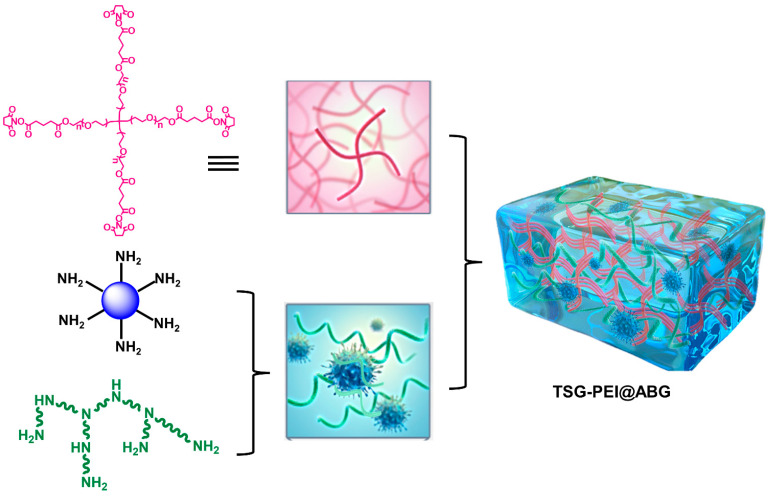
Schematic illustration of the fabricated TSG-PEI@ABG composite hydrogels.

Herein, we designed and prepared an inorganic–organic TSG-PEI@ABG hydrogel via the highly efficient ammonolysis reaction between (NHS)-ester of tetra-PEG-SG and branched PEI polymers in the presence of ABG particles in one-pot step (Figure 1). Covalent bonds rendered the TSG-PEI@ABG hydrogel with flexible injectability, fit-to-shape capacity, strong mechanics, and adhesiveness. Due to the reliable integration between soft network and rigid ABG particles on the nanoscale level, the facilitating adhesive strength and osteogenic performance were simultaneously achieved, indicating the hydrogel’s long-term stability and durable osteogenesis for the therapy of bone-related disease.

## 2. Materials and Methods

### 2.1. Materials

Tetra-arm poly(ethylene glycol) (tetra-PEG-OH, M_w_ = 20 kDa) was purchased from SINOPEG, China. Glutaric anhydride (98%), dimethylamino-pyridine (DMAP, 98%), and N-hydroxysuccinimide (NHS, 98%) were purchased from Energy Chemical Co., Ltd., Shanghai, China. 3-aminopropyltriethoxysilane (APTES, 98%) was purchased from J&K, Beijing, China. Branched polyethyleneimine (PEI, M_W_ = 1200, 99%) was purchased from Alfa Aesar. Bioactive glass (BG, Biological Grade) was purchased from Aladdin. All other chemical reagents were purchased from Energy Chemical Co., Ltd., Shanghai, China, and used as received without further purification.

### 2.2. Synthesis of Tetra-PEG-SG Polymer

First, tetra-PEG-OH (4 g, 0.2 mmol), glutaric anhydride (228 mg, 2 mmol), and DMAP (244 mg, 2 mmol) were dissolved in 50 mL of anhydrous DCM. After continuously stirring for 24 h, the DCM solution was washed with 2 M HCl solution, saturated NaCl solution, and DI water three times, followed by drying over the anhydrous MgSO_4_. The obtained product was purified by precipitating into the diethyl ether for several times to afford the intermediate of tetra-arm PEG-glutaric acid (tetra-PEG-COOH) polymer. Then, tetra-PEG-COOH (1 g, 0.05 mmol), EDCI (96 mg, 0.5 mmol), and NHS (58 mg, 0.5 mmol) were dissolved in 25 mL of anhydrous DCM. After continuously stirring for 24 h, the DCM solution was washed with 2 M HCl solution, saturated NaCl solution, and DI water three times and was followed by drying over the anhydrous MgSO_4_ to afford the white solid of tetra-PEG-SG polymer under the vacuum.

### 2.3. Synthesis and Modification of ABG Particles

The modification method to prepare ABG particle was performed according to the previous works [41,42]. Briefly, 0.3 g of BG and 5 mL of APTES were added into the hexane by continuously stirring at 60 °C. After 24 h, the ethanol and DI water were used to wash impurities several times, and the amine-terminated ABG particles were obtained after the drying process under the vacuum.

### 2.4. Preparation of TSG-PEI and TSG-PEI@ABG Hydrogels

TSG-PEI hydrogel was easily prepared after mixing the 15 wt% of tetra-PEG-SG and 1 mg/mL of PEI solutions at 37 °C without adding any other additive crosslinkers. As for the preparation of TSG-PEI@ABG hydrogel, the ABG was added into the PEI solutions in advance with continuous sonication for 30 min, and then mixed with tetra-PEG-SG solution via vortexing at 37 °C to achieve the gelation.

### 2.5. Nuclear Magnetic Resonance Spectra

Nuclear magnetic resonance (^1^H NMR and ^13^C NMR) spectra were obtained on a Bruker DRX-400 spectrometer (Bruker, Bremen, Germany) using the deuterated chloroform (CDCl_3_, δ = 7.26 ppm) as the solvent and tetramethyl-silane (δ = 0 ppm) as the internal standard.

### 2.6. Scanning Electron Microscopy

The network architecture of hydrogels was observed using scanning electron microscopy (SEM). Firstly, the hydrogels were freeze-dried to obtain the dry samples, and then a thin gold layer of Pt was sputter-coated onto the sample surface for 90 s to make them conductive. After that, the hydrogel networks could be observed via a JEOL JSM-6700F microscope with an acceleration voltage of 10 kV.

### 2.7. Compressive Measurement

The compressive strength of hydrogels was measured using a universal tensile machine (3365, Instron, Norwood, MA, USA) at a compressive speed rate of 2 mm/min. The hydrogel samples were cut into cylinders (diameter of 15 mm and height of 8 mm) for the measurement.

### 2.8. Rheological Measurement

The rheology behavior of hydrogels was conducted using a rheometer (Thermo Haake Rheometer, Newington, NH, USA). The measured hydrogel samples were spread on a parallel plate (25 mm) and sealed with silicone oil. A dynamic frequency scan in the range from 0.1 to 100 rad/s was used to record the storage and loss moduli (G′ and G″). The stress amplitude was set as 0.1% and the temperature was set at 25 °C.

### 2.9. Adhesive Study

Adhesion measurement was performed using the lap shear test by injecting 1 mL of gels onto the porcine skins, and commercially available fibrin glue was used as the control group. Briefly, standard lap shear test was performed on porcine skins that were adhered using the TSG-PEI, TSG-PEI@ABG, and fibrin glue for 30 s of pressing under a 200 g weight at room temperature before the tests, respectively. The porcine skins were cut into slices with a length of 60 mm, thickness of 10 mm, and width of 20 mm for the usage. All tests were performed with the universal testing machine (3365, Instron, Norwood, MA, USA) at a constant rate of 10 mm/min. The experiments were carried out at room temperature with a humidity of 45%, and each sample was tested five times to obtain the average values.

### 2.10. Cell Extraction and Culture

The bone marrow mesenchymal stem cells (BMSCs) were isolated from 3-month-old New Zealand white rabbits. The original generation of BMSCs was expanded up to passage 3 for experiments with the culture medium. The cells needed to be cultured in the medium and refreshed every 2 days in an environment of 37 °C and 5% CO_2_. Isolation, culture, trilineage differentiation potential assay, and immunophenotypic identification of BMSCs were verified as previously described in the literature [43].

### 2.11. In Vitro Cytotoxicity Assay

The cytotoxicity was performed using CCK-8 assay. The hydrogel extracts were prepared by placing the hydrogel into Dulbecco’s Modified Eagle’s medium (DMEM) at 37 °C for 24 h and then sterilizing them by filtration with a 0.22 μm filter. BMSCs were suspended in cell culture medium and seeded into 48-well plates with a density of 1 × 10^4^ cells/100 µL and incubated for 24 h at 37 °C in a 5% CO_2_ humidified incubator. The culture medium was then replaced with the hydrogel extract and incubated for another 24 h. Cells cultured in fresh medium were set as the control group. The cell viability (%) was calculated via the following equation:

Cell viability (%) = [(A_sample_ − A_blank_)/(A_control_ − A_blank_)] × 100%

### 2.12. Cell Proliferation Assay

Cell proliferation was also measured using CCK-8 assay. Firstly, the BMSCs were incubated in growth medium for 1 day, and then the hydrogel extracts were added into the medium and further incubated for another several days. After 7 days of incubation, cell culture medium was replaced with 100 µL of fresh culture medium and 10 µL of CCK-8 were added for another 4 h. Then, the absorbance at 450 nm was recorded on a microplate reader (Thermo, Waltham, MA, USA). Cell number was correlated with OD value for calculating the cell proliferation.

### 2.13. Live/Dead Staining Assay

Cell biocompatibilities were evaluated using a live/dead viability kit. The BMSCs after culture with hydrogel extracts was stained with live/dead staining working solution (Calcein-AM/PI) according to the manufacturer’s protocol. After culturing for 1 day, the stained cells were directly observed under the inverted optical microscope (Olympus, Tokyo, Japan). Calcein-AM generates a green fluorescence signal in living cells and PI dye only affects the nuclei of dead cells to emit a red fluorescence light. The number of live cells was quantitatively analyzed by Image J 1.8.0 software (Image J2, Wayne Rasband, Bethesda, ML, USA).

### 2.14. Alizarin Red S (ARS) Staining

BMSCs were seeded on the hydrogel extracts with a density of 1 × 10^6^ cells/well in osteogenic differentiation medium (Sigma, Springfield, MO, USA) including α-MEM supplemented with 10% of FBS, 1% of antibiotics, 50 μM of ascorbic acid, 10 mM of β-glycerol phosphate, and 0.1 μM of dexamethasone. After the osteogenic incubation for 7 and 14 days, the cells were washed three times, fixed with 4% of paraformaldehyde for 15 min, and stained for 30 min using the ARS staining kits at room temperature. The stained BMSCs were dried, and image J 1.8.0 software (Image J2, USA) was utilized to calculate the stained areas for semi-quantitative analysis.

### 2.15. Semiquantitative RT-PCR

The total RNA was extracted using the typical TRIzol Reagent and cDNA was synthesized from 200 ng of total RNA by RevertAidTM H Minus First Strand cDNA Synthesis Kit (Thermo Scientific, Waltham, MA, USA). Template PCRs were performed after 33 cycles of amplification with the adjustment annealing temperature. The primer sequences were listed as follows in Table 1.

### 2.16. Statistics Analysis

All the results were presented as the mean and standard deviation of 3–6 independent experiments. The statistics were analyzed by SPSS 22.0 software (SPSS Inc., Chicago, IL, USA). One-way ANOVA was used for more than two groups. *p* < 0.05 was considered to be significant.

## 3. Results

### 3.1. Preparation and Characterization of TSG-PEI and TSG-PEI@ABG Hydrogels

**Figure 2:**
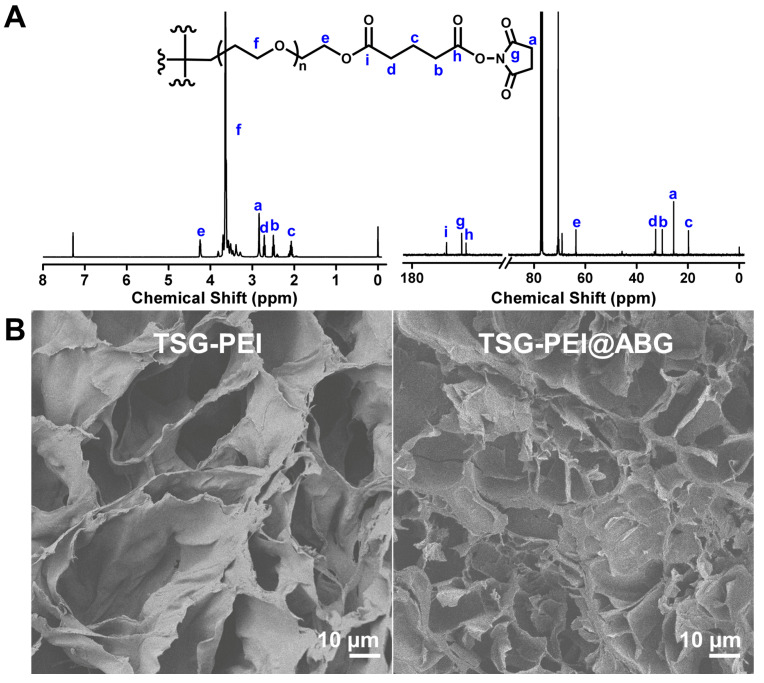
(**A**) ^1^H NMR and ^13^C NMR spectra of the tetra-PEG-SG polymer in CDCl_3_. (**B**) SEM images of TSG-PEI and TSG-PEI@ABG hydrogels.

The scheme illustration of preparation process of TSG-PEI@ABG hydrogel is shown in Figure 1. The tetra-PEG-SG polymer could be feasibly prepared via the two efficient reactions in mild conditions. The ^1^H NMR spectrum shows the explicit attribute of the characteristic peaks of tetra-PEG-SG with a very close integration ratio (a:b:c:d = 2:1:1:1) in Figure 2A. In addition, ^13^C NMR spectrum also clearly verified the structural integration and further proved the successful preparation of targeted polymer. Taking into consideration the abundant amine groups in the branched PEI polymers and ABG particles, the NHS-reactive tetra-PEG-SG polymer can be quickly reacted to form the TSG-PEI or TSG-PEI@ABG hydrogel networks after mixing them together, within seconds. It was mentioned that the ABG particles should be mixed with PEI solution under the sonication in advance to achieve the uniform arrangement as far as possible between the inorganic phases and organic materials. As shown in Figure 2B, the TSG-PEI hydrogel possessed interconnected pores that were benefited for the nutrition/metabolic waste exchange and cell adhesion, spreading and entry for both in vitro and in vivo experiments. After loading the rigid ABG particles into the soft polymeric networks, it was clearly observed that TSG-PEI@ABG hydrogel also exhibited suitable architectures and many pores for supporting the sustainable ABG release, cell adhesion, and substance exchange, but the deeper crosslinking with organic moieties could lead to denser networks and stronger mechanics.

**Figure 3:**
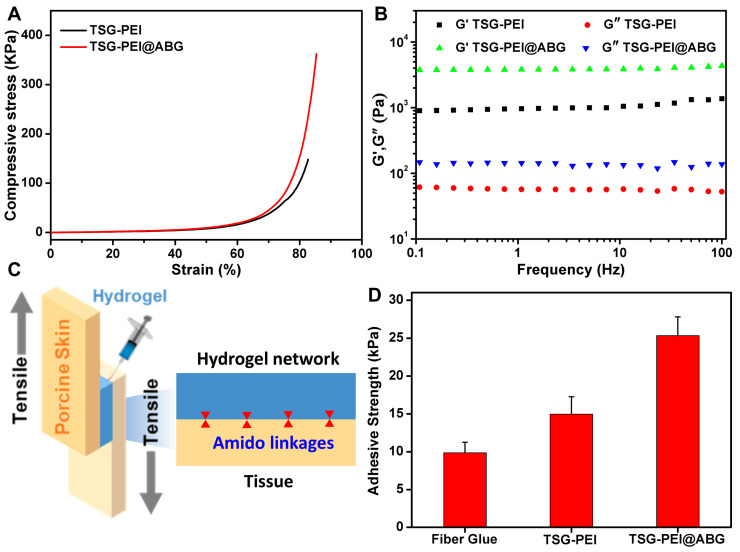
(**A**) Compressive and (**B**) rheological behaviors of TSG-PEI and TSG-PEI@ABG hydrogels. (**C**) Schematic illustration of the tissue adhesion test. (**D**) The tissue adhesion strengths of commercial fibrin glue, TSG-PEI, and TSG-PEI@ABG hydrogels.

Compared to the TSG-PEI hydrogel, the mechanical performances of TSG-PEI@ABG hydrogel were significantly improved in Figure 3. For example, the compressive strength and modulus of TSG-PEI@ABG hybrid hydrogel were more than twice those of the organic hydrogel, which indicated the uniform distribution of inorganic ABG particles within the hydrogel networks (Figure 3A). Similarly, the rheology testified the viscoelastic behavior of prepared hydrogels in the whole frequency and also elucidated that the introduction of ABG moieties significantly improved the mechanical modulus of the TSG-PEI@ABG hydrogel (Figure 3B), further demonstrating the rationalized networks and optimized structures of hybrid hydrogels. In addition, the greater storage modulus G′ and G″ further confirmed higher crosslinking density of the TSG-PEI@ABG hydrogel and suggested its potential key roles in the fabrication of implanted scaffolds for the nonload-bearing bone tissue repair.

For assessing one of the key factors to endow the engineered scaffold with high stability and durability during the practical application, the adhesive property of the hydrogel was quantitatively characterized on porcine tissue by a lap shear test (Figure 3C). Previous studies revealed that the adhesive performance of an adhesive was determined by the interface adhesive and mechanical strength of the hydrogel [44,45]. The interface adhesive strength was mainly dependent on the chemical reaction of the remaining NHS ester groups with tissue amines. As shown in Figure 3D, both the TSG-PEI and TSG-PEI@ABG hydrogels exhibited higher adhesion strength than the commercially available and clinical fibrin glue. Compared to the physical interaction of fibrin glue, the stronger tissue adhesion of prepared hydrogels was due to the rapid formation of chemical linkages among the amine-terminated proteins tissue and the tetra-PEG-SG polymers. It was mentioned that since hybridized ABG particles also possessed abundant amine groups, it could be integrated into an organic network via the physical aggregation effect and chemical ammonolysis reaction with ester-active tetra-PEG-SG polymers, spontaneously enhancing the adhesion and cohesion effects and forming a highly integrated inorganic–organic hybrid structure. Under this circumstance, ABG introduction endowed the hydrogel with powerful cohesion strength to satisfy the maintenance of durable stability and toughness onto the tissues, thus surpassing the weak strength of the TSG-PEI hydrogel. Therefore, the conventional hybridization method with BG moieties and elaborate optimization of involved ABG arrangement within the networks not only furnished the hydrogel scaffold with competitive adhesion and flexibility but also contributed to the tissue bioactivity in stimulating the osteogenesis and angiogenesis at the target sites, which is crucial for maintaining the long-lasting stability and bioactive osteogenesis in clinical bone repair applications.

### 3.2. Cell Viability and Proliferation

**Figure 4:**
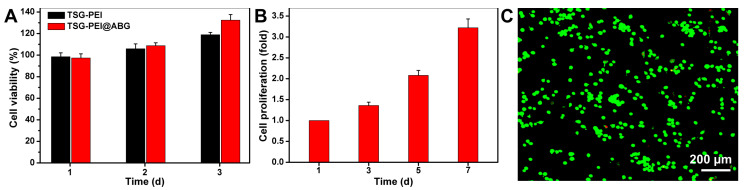
(**A**) Cell viability and (**B**) cell proliferation were detected by Cell Counting Kit-8 after cultivation for various times. (**C**) Live/dead staining of BMSCs after 1 day of culture. Green cells manifest living BMSCs and red cells manifest dead ones.

In view of its favorable structures, pores, mechanics, and adhesion force of the TSG-PEI@ABG hydrogel, it is necessary to assess the cell biocompatibility for this engineered scaffold. Therefore, we co-cultured the BMSCs with the hydrogel extract to evaluate the in vitro cytocompatibility by CCK-8 and live/dead staining assays. Quantitatively, the TSG-PEI hydrogels had capacities on maintaining a high cell survival level over the times with or without being laden with ABG. The cell viability ratio at day 3 was obviously higher than that of day 1, indicating the low cytotoxicity and high cell proliferation behaviors (Figure 4A). In addition, the ABG intervention not only did not impair the material cytotoxicity but also promoted the cell growth, which indicated the uniform dispersion within the networks instead of the large aggregates and implied the important roles of ABG substrate on promoting cell proliferation after prolonged incubation. As expected, the cells within the TSG-PEI@ABG hydrogel continued to increase after 7 days of culture in vitro (Figure 4B), further revealing the outstanding cell activity and proliferation behavior of the ABG-doped hybrid scaffold. Live/dead assay in Figure 4C showed almost all the luminous green lights and clearly demonstrated the high cell viability after culture with this hydrogel scaffold for 24 h. Therefore, we believe that the porous structures and good mechanical properties of this biocompatible TSG-PEI@ABG scaffold are able to enhance cell activity, growth, proliferation, and differentiation.

### 3.3. Osteogenic Differentiation of BMSCs in the Hydrogel Scaffolds In Vitro

**Figure 5:**
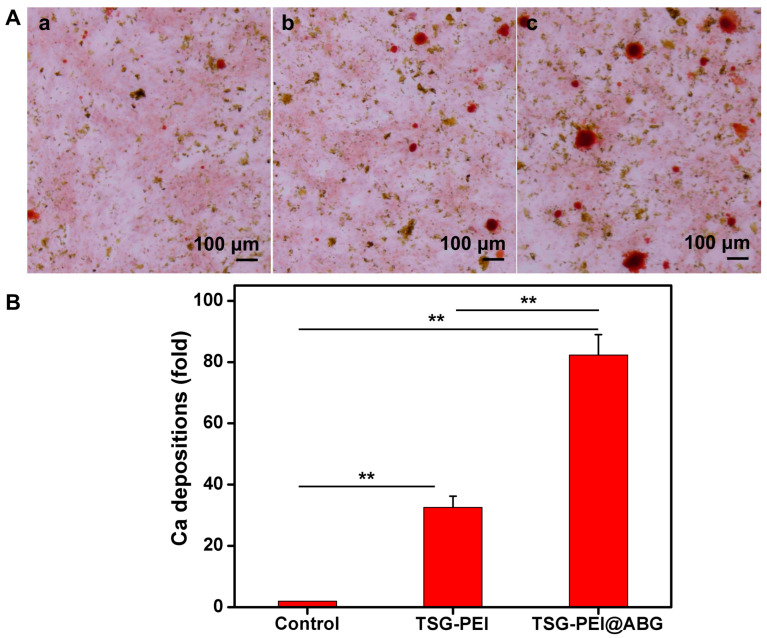
(**A**) Osteogenic differentiation detected based on Alizarin red. (**a**) The control (untreated cells), (**b**) TSG-PEI hydrogel, (**c**) TSG-PEI@ABG hydrogel. (**B**) In vitro osteogenic differentiation via the semi-quantification of calcification depositions. Statistically significant differences in comparison with control untreated cells, TSG-PEI hydrogel, and TSG-PEI@ABG hydrogel (** *p* < 0.01).

To reveal the effect of hydrogel scaffolds on the osteogenic differentiation in vitro, we seeded BMSCs cells on the control, TSG-PEI, and TSG-PEI@ABG hydrogel scaffolds. ARS staining and qPCR assay were used to examine the osteoinduction capacity. As shown in Figure 5, the TSG-PEI@ABG hydrogel scaffold could conspicuously increase the calcification nodule formation of BMSCs after 14 days of osteogenic induction, surpassing the other two groups in terms of both quantity and volume, thus demonstrating that the BMSCs cultured on this hydrogel scaffold had obvious activation and enhanced the osteogenic differentiation.

**Figure 6:**
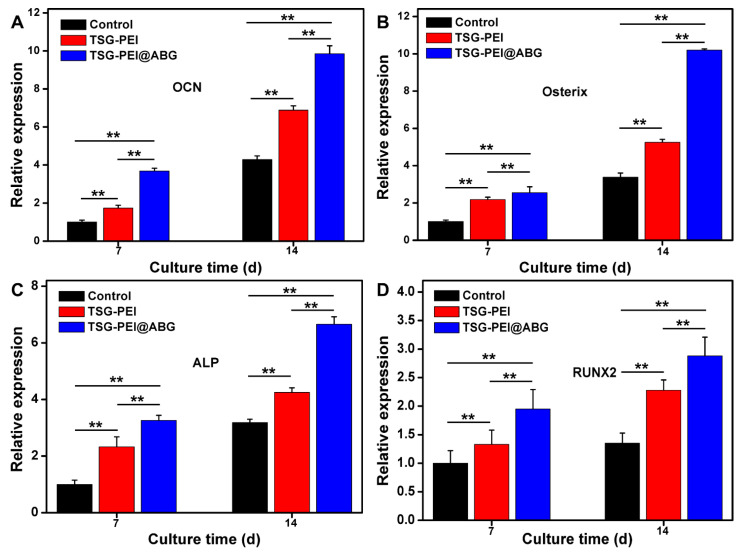
qRT-PCR analysis of the mRNA levels of relative gene expressions of (**A**) OCN, (**B**) Osterix, (**C**) ALP, and (**D**) Runx 2 on days 7 and 14 of culture. Statistically significant differences of the control group, TSG-PEI group, and TSG-PEI@ABG group (** *p* < 0.01).

In addition, the mRNA levels of expression of the osteogenesis-related marker genes of OCN, Osterix, ALP, and Runx 2 were investigated using the control, TSG-PEI, and TSG-PEI@ABG hydrogels, as shown in Figure 6. On day 7, real-time PCR results showed that all the four key osteogenic markers in the TSG-PEI@ABG group were significantly upregulated compared to those in the control group. It was mentioned that the higher gene expressions in the TSG-PEI@ABG hydrogel than those of TSG-PEI hydrogel indicated the improved mechanical properties and promoted osteogenic differentiation performances due to the introduction of bioactive ABG moieties and contribution of denser hybrid networks. On day 14, these levels of the TSG-PEI@ABG group were significantly higher than those of the TSG-PEI and control groups, further suggesting that the ABG introduction could effectively promote osteogenic differentiation of stem cells for a long period in vitro. During the culture periods, ABG particles may be expose-contacted into the stem cells to favor the angiogenesis because of their biological components of silicon, calcium, and phosphorus elements, which can play important roles in facilitating the bone mineralization and eventually enhancing the bone regeneration along with the gradual hydrogel degradation in vivo [41,42].

## 4. Conclusions

In summary, we constructed a kind of hybrid hydrogel adhesive via the efficient NHS–amine chemistry due to the availability and affordability of the polymeric components of NHS-activated tetra-PEG-SG polymer and the amine groups of branched PEI polymer and ABG particles. Under the high efficiency of the crosslinking reaction, the obtained TSG-PEI@ABG hydrogel displayed suitable porous architectures, sufficient compressive and adhesive strength, and facilitated a moisture environment for the stem cell survival, growth, and proliferation. It was particularly stressed that NH_2_-modified ABG introduction not only contributed to the hybrid crosslinking junction to enhance the cohesive strength and structural stability, but also provided the crucial osteogenic components to guide the osteogenesis. Consequently, compared to previous attempts at preparing the engineered hydrogel scaffolds for osteogenesis, the current attempt has put forward an adaptive hybridization strategy, offering a potential candidate with feasible usage availability and robust osteogenic potency.

## Figures and Tables

**Table 1:** The primer sequences used for the RT-PCR.

Gene	Forward Primers (5′–3′)	Reverse Primers (5′–3′)
*GAPDH*	AGGTCGGTGTGAACGGATTTG	TGTAGACCATGTAGTTGAGGTCA
*RUNX2*	AGTGACTGGGAAACCAGATGCTGA	GCTCTTGGCAAATCTGGCGTGTAA
*ALP*	CCAACTCTTTTGTGCCAGAGA	GGCTACATTGGTGTTGAGCTTTT
*OCN*	CTGACCTCACAGATCCCAAGC	TGGTCTGATAGCTCGTCACAAG
*Osterix*	ATGGCGTCCTCTCTGCTTG	TGAAAGGTCAGCGTATGGCTT

## Data Availability

Not applicable.
